# Grade IV traumatic pancreatic injury with primary duodenum malignant lymphoma following pancreatoduodenectomy: a case report

**DOI:** 10.1186/s40792-020-00817-w

**Published:** 2020-03-18

**Authors:** Kosuke Ono, Tomoyuki Abe, Hironobu Amano, Shuji Yonehara, Tsuyoshi Kobayashi, Masahiro Nakahara, Hideki Ohdan, Toshio Noriyuki

**Affiliations:** 1 Department of Surgery, Onomichi General Hospital, Onomichi, Hiroshima Japan; 2 Department of Pathology, Onomichi General Hospital, Onomichi, Hiroshima Japan; 3 Department of Gastroenterological and Transplant Surgery, Graduate School of Biochemical and Health Sciences, Hiroshima University, Hiroshima, Japan; 4 Department of Surgery Endoscopic Surgery, Onomichi General Hospital, Hirahara 1-10-23, Onomichi, Hiroshima 722-8508 Japan

**Keywords:** Pancreatoduodenectomy, Follicular lymphoma, Duodenum, Elderly, Trauma, Pancreatic injury

## Abstract

**Background:**

Traumatic pancreatic injury with a main pancreatic duct injury has a high incidence of mortality and requires a prompt and appropriate treatment. However, the best approach, and treatment options, which may be limited, remains controversial especially for the elderly patients. Herein, we present a case of traumatic pancreatic injury in an elderly patient for whom pancreatoduodenectomy was safe and effective.

**Case presentation:**

An 87-year-old man was diagnosed with a traumatic pancreatic injury with a main pancreatic duct injury. In addition, the horizontal segment of the duodenum was largely eradicated. There were no comorbidities, and his vital signs were stable. A pancreatoduodenectomy was performed. The postoperative course was uneventful, and he was discharged. Pathological examination revealed a primary follicular lymphoma of the duodenum.

**Conclusions:**

This case demonstrated that pancreatoduodenectomy could be performed safely for a severe pancreatic injury in an elderly patient. However, special attention should be paid to select the optimal surgical procedure. Further, this was a rare case, as initially a primary follicular lymphoma of the duodenum was suspected as a duodenal injury coexisting with a traumatic pancreatic injury because of the increased duodenal thickness.

## Background

Traumatic injuries to the pancreas are relatively rare. In addition to their rarity, the anatomical location renders it difficult to select an optimal treatment [1, 2]. Among blunt abdominal injuries, pancreatic injury is highly associated with neighboring organ injuries, and even after a curative surgical intervention, the outcome is often not satisfactory [3]. Due to advances in treatment strategies for blunt organ injuries, including the liver, kidney, and spleen, nonoperative management (NOM) has become the standard of care under strict eligibility criteria [4]. The most commonly used injury grading system for pancreatic injuries is the Organ Injury Scale of the American Association for the Surgery of Trauma (AAST-OIS), which describes the anatomical site of the lesion on the pancreas and status of the main pancreatic duct [5]. For injuries of AAST-OIS grades I and II, the outcomes of NOM are superior to those of the surgical intervention [4]. For AAST-OIS grade V injuries, in which there is a devastating injury to the head of the pancreas, pancreatectomy is considered inevitable. However, for injuries of AAST-OIS grades III and IV, the best practice remains controversial [6, 7]. In these grades, operative management is necessary; however, there is a lack of robust evidences of data that support pancreatic resection versus drainage only. Herein, we present a case of a grade IV traumatic injury to the pancreas in an elderly patient for whom pancreatoduodenectomy (PD) was safe and effective.

## Case presentation

**Fig. 1:**
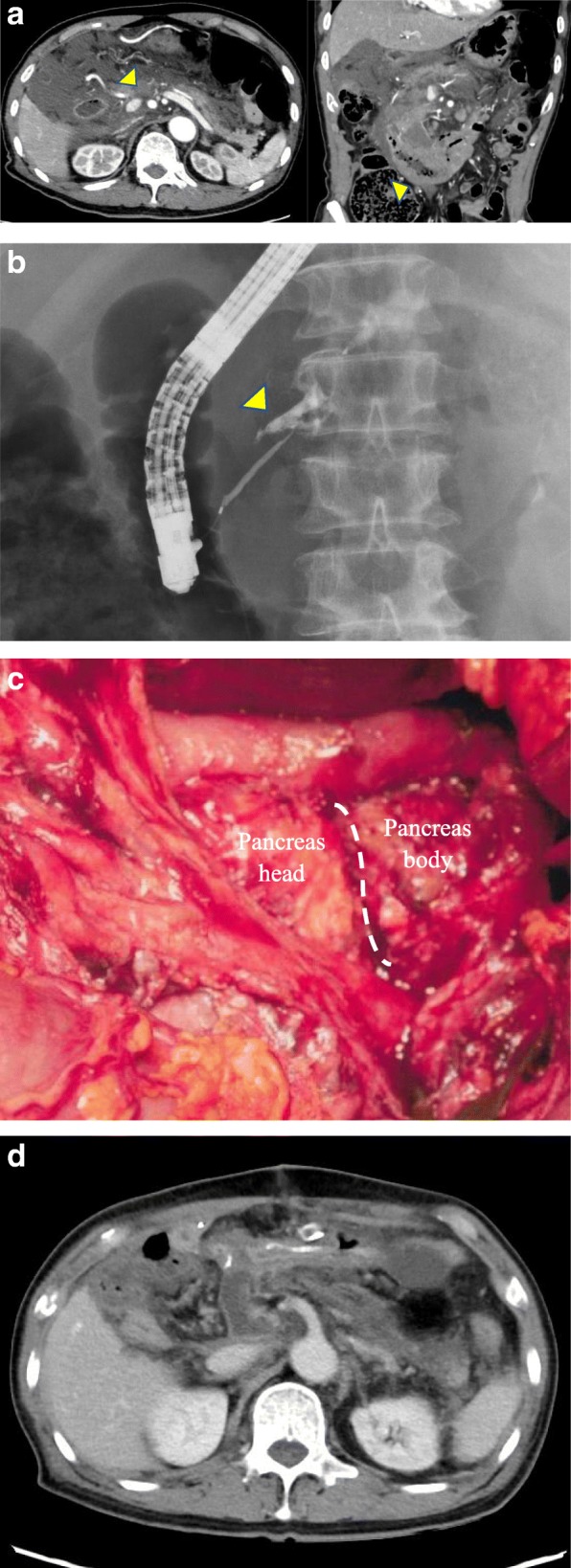
**a** Abdominal contrast-enhanced computed tomography. Fluid retention around the pancreas (arrow) and wall thickening of the horizontal segment of the duodenum (arrow) are observed. **b** Pancreatography. Massive leakage of the contrast medium from the main pancreatic duct at the pancreatic head (arrow) is observed. **c** Pancreas head and body were completely transected. **d** Abscess formation or pseudoaneurysm formation were not found

An inherently healthy 87-year-old man underwent first aid after a traffic accident. At the hospital, his general condition was stable and there were no abnormalities in his vital signs. Blood biochemistry tests showed an increased inflammation and a marked increase in pancreatic enzymes (AMY 818 U/L, P-AMY 705 U/L, and lipase 1191 U/L). Abdominal contrast-enhanced computed tomography showed fluid retention around the pancreas and wall thickening of the horizontal segment of the duodenum (Fig. 1a). The pancreatic head was severely injured, and laceration to the pancreatic head was suspected. The duodenum wall was enlarged, especially at the third part, and the horizontal segment was considerably large. Endoscopic retrograde cholangiopancreatography was performed to evaluate the presence of a main pancreatic duct injury. Pancreatography revealed a substantial leakage of the contrast medium from the main pancreatic duct at the pancreatic head (Fig. 1b). Endoscopic naso-pancreatic drainage (ENPD) was performed at the proximal side of the injury.

Following the diagnosis of a pancreatic injury of grade IV or V, an emergency operation was performed. Intraoperative findings confirmed the severe pancreatic head injury (Fig. 1c). The pancreas was completely transected at the head of the pancreas, but the degree of disruption itself was not high. Based on these findings, the present case was classified as grade IV according to the AAST-OIS classification. In addition, the horizontal segment of the duodenum was widely swollen and its wall was thickened.

**Fig. 2:**
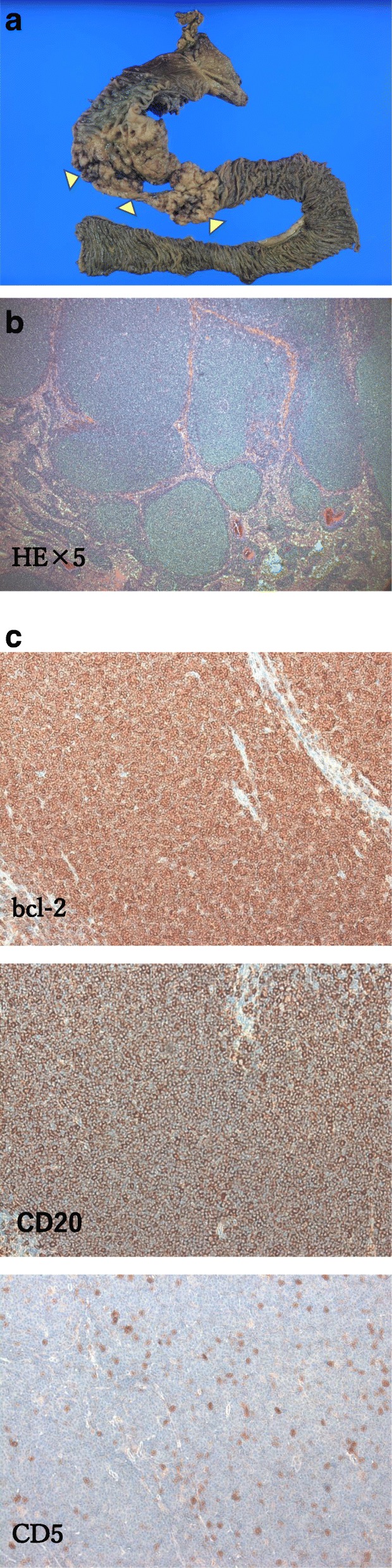
**a** Mucosal enlargement and irregular bumps were found from the descending segment to the horizontal segment (arrow). **b** Histological examination. Atypical lymphocytes with a sickle-shaped nucleus are observed in a nodular structure in the area of duodenum wall thickening (hematoxylin and eosin staining, × 5). **c** Immunohistochemical staining. Staining is positive for CD20 and bcl-2 and negative for CD5

The ENPD tube could not be differentiated from the main pancreatic duct because the ENPD interposition was moved by the operative procedure. Our first treatment strategy was a primary repair by suturing the pancreatic laceration. However, the ENPD tube could not be detected at the main pancreatic duct, and the severe laceration would not heal by simple suturing. Although damage control surgery would have been another choice for this condition, our suspicions of a massive duodenum injury required us to remove this injured organ. We used PD-II as the reconstruction method. Pancreatojejunostomy was performed, with the Blumgart modification added for pancreatic duct-to-mucosa anastomosis. The pancreas was transected diagonally, and it was difficult to use the lacerated pancreas in anastomosis, so an anastomosis was performed by adding a pancreatic resection on the distal side. The operative time was 389 min, and blood loss was 527 mL. There were no postoperative events. No abnormal findings such as an abscess formation or pseudoaneurysm formation were found on computed tomography images 10 days after surgery (Fig. 1d). He was discharged on postoperative day 25. Examining the resected specimen of the pancreas, the pancreatic parenchyma revealed acute pancreatitis with an evidence of fat necrosis and neutrophil infiltration into the pancreatic parenchyma. The resected specimen of the duodenum also revealed that a mucosal enlargement and irregular bumps were found from the descending segment to the horizontal segment (Fig. 2a). Microscopic findings showed that atypical lymphocytes with a sickle-shaped nucleus and a nodular structure in the segment of the duodenum wall thickening (Fig. 2b) occurred. Immunohistochemical staining was positive for CD20 and bcl-2 and negative for CD5 (Fig. 2c). Duodenum follicular lymphoma (FL) was diagnosed.

## Discussions

The present case confirms pancreatoduodenectomy as an optimal treatment strategy under strict patient eligibility criteria, including stable circulatory dynamics and concomitant neighboring organ injury, even in elderly patients. Of note, the patient had a rare condition of a swollen duodenum from the second to the third segment that mimicked blunt trauma injury to the duodenum but was histologically diagnosed as primary FL of the duodenum. Definitive diagnosis is very difficult in cases of pancreatic injuries; close attention should be paid to other potential organ injuries to select the appropriate surgical procedure. The incidence of pancreatic injury has been reported to be less than 1% among cases of abdominal blunt trauma [8].

Optimal management of the pancreatic injury remains controversial for injuries of grades III or IV. As previously mentioned before, there are established opinions on the management policy for injuries of AAST-OIS grades I, II, and V. For grades III and IV injuries, surgical management is considered the basic optimal treatment. However, the type of surgical intervention has varied from drainage only, suturing repair, to pancreatic resection with or without immediate reconstruction [9]. Because the method cannot be easily selected, the probability of postoperative complications due to pancreatic resection or anastomosis in a pancreatic injury is as high as 40% [10]. Recently, some reports have suggested that NOM using endoscopic pancreatic duct stenting could be a useful and safe option in selected patients [11]. In this case, we selected PD because we suspected a co-existing duodenal injury. Due to its retroperitoneal location and proximity to the major vascular structures and other organs, isolated pancreatic injuries are rare [8, 12]. Thus, the wall thickening was suspected to reflect damage by the injury. However, the duodenal wall thickening was, in fact, due to primary duodenal FL, which was incidentally identified in the present case.

Primary gastrointestinal FL is rare, and among such cases, the frequency of primary duodenal FL is extremely rare at 3.6% [13]. Duodenal FL is mostly asymptomatic and often discovered incidentally by upper gastrointestinal endoscopy or diagnosed during a complication, such as a perforation [14]. To our knowledge, there is no report on FL incidentally identified because of a grade IV pancreatic injury. Immunostaining is useful for a definitive diagnosis, and FL can be diagnosed if bcl-2 is positive at the center of the lymphoid follicle. The prognosis of duodenal FL is better than that of other malignant lymphomas. An established treatment strategy has not been reported; “watch and wait” is currently acceptable for this slow-growing disease [15].

In the present case, suspicion of a widespread duodenal injury was the main reason for selecting pancreatoduodenectomy. Some reports have shown that in pancreatic injuries, pancreatoduodenectomy has high mortality (30%) and complication rates (80%) [16]. Furthermore, the risk of postoperative complications in pancreatoduodenectomy may be higher at age 75 years and older [17]. Thus, pancreatoduodenectomy is a very risky procedure, and adaptations should be carefully performed. With the aging society, the chances of encountering a case such as ours may increase. Highly invasive general abdominal surgeries, such as hepatectomy, are being applied to healthy elderly people [18]. Consistent with this, pancreatoduodenectomy might also be a useful treatment for elderly people with stable vital signs and suspected widespread duodenal injury.

## Conclusion

In conclusion, the present case demonstrated that pancreatoduodenectomy could be performed safely for grade IV pancreatic injuries in elderly patients under strict eligibility criteria such as stable vital signs, although our case was rare in that primary FL of the duodenum was suspected as duodenal injury coexisting with traumatic pancreatic injury because of the increased duodenal thickness.

## Data Availability

Not applicable

## Abbreviations

AAST-OIS: Organ Injury Scale of the American Association for the Surgery of Trauma
ENPD: Endoscopic naso-pancreatic drainage
FL: Follicular lymphoma
NOM: Nonoperative management
